# Can systematic implementation support improve programme fidelity by improving care providers’ perceptions of implementation factors? A cluster randomized trial

**DOI:** 10.1186/s12913-022-08168-y

**Published:** 2022-06-22

**Authors:** Miriam Hartveit, Einar Hovlid, John Øvretveit, Jørg Assmus, Gary Bond, Inge Joa, Kristin Heiervang, Bjørn Stensrud, Tordis Sørensen Høifødt, Eva Biringer, Torleif Ruud

**Affiliations:** 1 Valen Hospital Helse Fonna HF, 5451 Valen, Norway; 2 Department of Global Public Health and Primary Care, University of Bergen, Box 7804, 5020 Bergen, Norway; 3 Department of Social Science, Western Norway University of Applied Sciences, Røyrgata 6, 6856 Sogndal, Norway; 4 Stockholm Health Care Services, Region Stockholm (SLSO) and LIME/MMC, Tomtebodavägen 18A, Karolinska Institutet, Stockholm, Sweden; 5 Centre for Clinical Research, Haukeland University Hospital, Box 1400, 5021 Bergen, Norway; 6 Westat, Rivermill Commercial Center, 85 Mechanic Street, Lebanon, NH USA; 7 Network for Clinical Research in Psychosis, Stavanger University Hospital, Box 8100, 4068 Stavanger, Norway; 8 Network for Medical Sciences, Faculty of Health, University of Stavanger, Stavanger, Norway; 9 Division of Mental Health Services, Akershus University Hospital, Box 1000, 1478 Lørenskog, Norway; 10 Division of Mental Health, Innlandet Hospital Trust, Box 104, 2381 Brumunddal, Norway; 11 University Hospital Northern Norway, Box 6124, 9291 Tromsø, Norway; 12 Department of Research and Innovation, Helse Fonna HF, 5416 Stord, Norway; 13 Institute of Clinical Medicine, University of Oslo, Box 1171 Blindern, 0318 Oslo, Norway

**Keywords:** Implementation, Mental health, Readiness, Implementation process, Implementation outcomes

## Abstract

**Background:**

Investigations of implementation factors (e.g., collegial support and sense of coherence) are recommended to better understand and address inadequate implementation outcomes. Little is known about the relationship between implementation factors and outcomes, especially in later phases of an implementation effort. The aims of this study were to assess the association between implementation success (measured by programme fidelity) and care providers’ perceptions of implementation factors during an implementation process and to investigate whether these perceptions are affected by systematic implementation support.

**Methods:**

Using a cluster-randomized design, mental health clinics were drawn to receive implementation support for one (intervention) and not for another (control) of four evidence-based practices. Programme fidelity and care providers’ perceptions (Implementation Process Assessment Tool questionnaire) were scored for both intervention and control groups at baseline, 6-, 12- and 18-months. Associations and group differences were tested by means of descriptive statistics (mean, standard deviation and confidence interval) and linear mixed effect analysis.

**Results:**

Including 33 mental health centres or wards, we found care providers’ perceptions of a set of implementation factors to be associated with fidelity but not at baseline. After 18 months of implementation effort, fidelity and care providers’ perceptions were strongly correlated (B (95% CI) = .7 (.2, 1.1), *p* = .004). Care providers perceived implementation factors more positively when implementation support was provided than when it was not (t (140) = 2.22, *p* = .028).

**Conclusions:**

Implementation support can facilitate positive perceptions among care providers, which is associated with higher programme fidelity. To improve implementation success, we should pay more attention to how care providers constantly perceive implementation factors during all phases of the implementation effort. Further research is needed to investigate the validity of our findings in other settings and to improve our understanding of ongoing decision-making among care providers, i.e., the mechanisms of sustaining the high fidelity of recommended practices.

**Trial registration:**

ClinicalTrials.gov Identifier: NCT03271242 (registration date: 05.09.2017).

**Supplementary Information:**

The online version contains supplementary material available at 10.1186/s12913-022-08168-y.

## Background

Implementing new knowledge into existing practices has been a challenging task for health care services for many years [1]. To improve implementation success, we need more guidance on how implementation is accomplished and sustained [2, 3]. Most implementation efforts (i.e., clinical unit’s activities to implement a new practice in every daily practice) make use of data on implementation outcomes, such as fidelity scores, to monitor the progression and achievement of implementation [4]. Fidelity can be defined as the degree to which an intervention was implemented as it was described in the original protocol [2]. Fidelity scores provide information on the degree to which the new practice is implemented as designed [4]. However, these data cannot inform us about why the implementation progression is incomplete or how we should address these issues to improve implementation outcomes. The literature suggests several implementation factors, i.e., interrelated, coexisting moderators for implementation outcomes to explain implementation, such as collegial support and readiness [2, 5]. In mental health services, active leadership to redesign the workflow, measurement and feedback are found to be important implementation factors [6]. However, the evidence is limited and currently, there is a lack of reliable techniques to effectively address deficits in these factors once they are identified [5]. Evidence of implementation factors that are both associated with implementation outcomes and found to be affected by systematic implementation support is warranted to foresee challenges and effectively intervene based on these factors.

Few of the existing, theoretically founded, implementation factors have demonstrated reliable associations with implementation outcomes [2, 7–9]. There may be several methodological reasons for this apparent lack of association. First, many of the constructs are investigated individually rather than as a set of interrelated factors. The interdependency between factors implies that implementation success is determined by a set of potentially shifting factors [7]. Implementation should therefore be investigated as complex interventions, i.e., as a set of interacting factors [10, 11]. Second, many investigations are based on the assumption that sufficient preparedness prior to implementation implies success. Examples are organizational readiness for change [8, 12] and clinicians’ attitudes towards evidence-based practice [13]. However, stages of change theory by Prochaska, Rogers and others describe stages of orientation, insight and acceptance for making and maintaining a change, and these stages are iterative [14–16]. Care providers constantly assess pros and cons and may reconsider their involvement in the change. A more process-based approach to readiness than defining it as a pre-state is recommended [17]. Investigations of implementation factors solely before entering the active implementation phases do not consider the continuous exposure of contextual factors under which care providers decide whether they should continue implementation [18]. Third, many factors are investigated by observations. Examples are the model for understanding success in quality (MUSIQ) [19] and stages of implementation completion (SIC) [20]. External observation is not in line with theories that define alteration of behaviour as a social construct, i.e., an experience by those making the change. Examples are Agyris’ double loop learning and Rogers’ diffusion of innovation. They both emphasize the change agents’ sense of support, gains and obstacles, rather than objective observation, when explaining change in behaviour [14, 21]. Finally, there are methodological limitations to existing research on implementation factors. Most studies employ retrospective, uncontrolled designs or qualitative investigations [2, 22]. To reveal the relevance of implementation factors for fidelity and other implementation outcomes and whether systematic implementation support can impact these factors, we need a randomized controlled design with longitudinal data collections during implementation efforts. Randomised controlled designs have been employed to investigate the impact of implementation strategies on patient outcome, such as implementation of the Crisis resolution teams in mental health care [23]. However, we lack systematic investigations of the associations among implementation strategies, facilitating and hindering contextual factors, and implementation outcomes [24].

We aimed to assess the associations between fidelity and care providers’ perceptions of a set of implementation factors and investigate whether these perceptions can be affected by systematic implementation support. We wanted to investigate these associations and impacts at various timepoints in time during an implementation effort.

## Methods

Using a cluster-randomized controlled trial design, specialist mental health clinics were invited to implement recommended practices for the treatment of psychosis. Each clinic chose two of four predefined practices they intended to implement and was randomized to implementation support for one practice (intervention arm) and not for the other (control arm). We prospectively measured health professionals’ perceptions of the implementation effort and the clinics’ degree of implementation every sixth month during an 18-month period. This was measured for both the practice they received implementation support for and for the control in each clinic.

### Sample and randomization

This study was conducted within specialized mental health care in six Norwegian public health trusts (local health authorities) serving 38% of the country’s population in both rural and urban areas. Thirty-nine specialist mental health care clinics, i.e., 21 community mental health centres and 13 mental health hospital departments (e.g., forensic wards or specialist units for the treatment of psychosis) participated. They were invited in 2016 by the research group to take part in a study to improve compliance with the national clinical guidelines for the treatment of psychosis. Four evidence-based practices described in the guideline were chosen by the project group after a survey among the included mental health clinics. These were practices or programmes for antipsychotic medication management (MED), physical health care (PHYS), family psychoeducation (FAM), and illness management and recovery (IMR). Each clinic decided two of these four practices for implementation. Among the 39 clinics invited, 17 chose antipsychotic medication, 26 chose physical health care, 14 chose family psychoeducation and 21 chose illness management and recovery. Four unique fidelity scale were developed and employed by audit teams to score the degree of compliance to the core components of the practices the clinics had chosen. (The scales are further described under “Measures”).

By using pairwise randomization, we balanced the number of sites assigned to the intervention or control arms within each practice and ensured that each clinic was represented in both the intervention group and the control group. In the US National Evidence-Based Practice Project [25], the mean EBP fidelity increased from 2.28 (SD 0.95) at baseline to 3.76 (SD 0.78) at 12 months (See: Ruud et al. [26]). We assumed initially a similar mean increase in fidelity over 18 months for the experimental practices and no increase for control practices. Based on a two-tailed significance level of 5 and 90% power, we estimated that the overall hypothesis would be adequately powered with a minimum of eight sites in each arm for each practice.

To represent each clinic, a purposive sample of care providers, selected by their manager as particularly important for the clinical practice of psychosis treatment, constituted the sample of Implementation Process Assessment Tool (IPAT) responders. A sample of 10–12 care providers per clinic were suggested from the project, but the managers decided how many of their staff meet the inclusion criteria, resulting in five to 32 being invited per clinic. We decided a cut-off of three or more responders per clinical unit as an inclusion criterion for this study. The cut-off was set based on the logic of balancing generalizability to the unit and representation of a variety of clinical units. At the time of the first data collection (baseline), the clinics were recently informed about which practice they would receive implementation support for to enable preparations, such as deciding who should attend the clinical training.

### The intervention (implementation support)

**Fig. 1:**
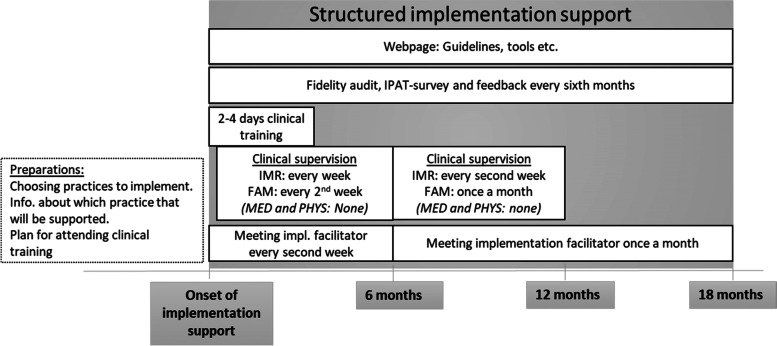
Description of the intervention: The structured implementation support included a set of interventions, here displayed on a timeline to show when and how long each intervention was provided for each of the four practices (*IMR *Illness Management and Recovery practice, *FAM *Family supportive practice, *MED *Antipsychotic medication, *PHYS *Physical health support)

The intervention bundle was designed to affect care providers’ readiness and engagement and included tools and measures described in the literature [25, 27] and more innovative measures. It consisted of clinical training, implementation facilitation, and assessment and feedback every sixth month on level of compliance to recommended care and health professionals’ anonymous report on how they perceived the implementation effort. The clinical training included workshops with lectures and meeting colleagues from across the country, lasting one day for medication and physical health practices and 2 × 2 days for IMR and family support practices. For the IMR practice and the family support practice, the clinicians were offered additional supervision by a clinical expert on the practice. For IMR, this was offered every week for the first six months and every second week for the next six months. For the family support it was offered every other week for six months and then monthly for the next six months. A website with further educational material and tools was made available for the four practices. The implementation facilitation included support and training to enhance positive perceptions of the implementation effort i.e., planning and using tools and methods such as flow charts, process measuring and team activities to improve shared understanding and accountability. The clinics were offered one meeting with trained implementation facilitators every second week for the first six months and then once a month for a year. The implementation facilitators were trained by implementation experts in the project to recognize and interpret care providers’ perceptions of the factors facilitating and hindering implementation, measured by the IPAT questionnaire, and to suggest tools and methods to improve these perceptions and thereby make implementation teams successful. They had relevant clinical experience and training but were not clinical experts on any of the four practices to be implemented. The intervention included assessments and feedback on the clinic’s degree of compliance with the practice they had chosen and received support for, measured by the fidelity scale. Additionally, feedback on implementation factors, i.e., clinicians’ perceptions of the implementation effort as measured by the IPAT questionnaire, was provided. Fidelity assessments and the IPAT were delivered every six months over the 18-month intervention period. The feedback on IPAT scores was provided to the manager, the implementation team and the implementation facilitator at each clinic shortly after each measurement together with a short report to help interpret and suggest interventions to improve low scores. Please see Fig. 1. This intervention bundle to support implementation was in an earlier paper from this trial shown to have a positive impact on the fidelity scores. We found the difference between experimental and control sites in mean increase in fidelity score (within a range 1–5) over 18 months was 0.86 with 95% CI (0.21–1.50), *p* = 0.009, with corresponding effect size 0.89 (95% CI 0.43–1.35) [26].

### Measures

The implementation was assessed using a specific fidelity scale for each of the four evidence practices. The four fidelity scales are the Physical Health Care Fidelity Scale [28], the Antipsychotic Medication Management Fidelity Scale [29], the Family Psychoeducation Fidelity Scale [30], and the Illness Management and Recovery Fidelity Scale [31]. Each fidelity scale contains 13–17 items for rating core components of the practice and detailed criteria for rating each item from 1 (lack of adherence to the evidence-based model) to 5 (full adherence to the model). The total fidelity score is calculated as the unweighted mean score of the items in the fidelity scale. The fidelity scales and their psychometric properties are further described in earlier papers [28–31]. An assessment team of two clinicians trained to use the fidelity scales conducted the scoring. At each clinic, they interviewed clinicians and managers, reviewed documents (e.g., procedures and data on performance) and conducted audits of ten randomly selected patient records for patients receiving psychosis treatment. The 13–17 items of the fidelity scales were rated on a scale from 1 (not implemented) to 5 (perfectly implemented). The unit of analysis for fidelity score is the clinic, defined by the mean of the items.

Health professionals’ perceptions of the implementation factors were measured by the IPAT [32]. The IPAT questionnaire is theoretically based on constructs defined in the Consolidated Framework for Implementation Research [33], including readiness for change [34] and stages of change [14, 15, 35]. In the theoretical model for the IPAT construct, outer- and inner-setting factors, intervention characteristics and the characteristics of individuals are perceived and interpreted at the individual and team levels. The implementers react to their perceptions by proceeding (or resisting and reversing) on the stages of change continuum from *insight* to *acceptance* and *sustainment*. This progression, in turn, affects how the implementers perceive the implementation effort [32]. The 27 items measure progression in the stages of change, individual activities and perceived support, collective readiness and support, and individual perception of the intervention. Examples of items are “*I have discussed with colleagues how this new practice will work in our unit”,*” *I believe the patient will benefit from the improvement” and “We who work here agree that we have a potential for improvement in [new practice]”.* The questionnaire was distributed to a purposive sample of health professionals central to the provision of care in question. The Likert scale from 1 (not agree/not true) to 6 (= agree/correct) for each of the 27 items revealed variation between respondents and between clinics. The internal consistency for the total scale and for each of the four underlying constructs was found to be high (Cronbach’s alpha >.83) [32]. The IPAT’s unit of analysis is the clinical unit and is represented by the mean of the responders from the clinic.

Fidelity and IPAT were scored for the practice in which implementation support was provided and for the control practice in each clinic at baseline, 6 months, 12 months and 18 months.

### Analyses

Descriptive methods (mean, percent and standard deviation) and confidence intervals were used to describe the IPAT responders and the IPAT and fidelity scores. The associations between care provider perceptions (IPAT scores) and fidelity scores at each time point were explored by linear mixed effects models for fidelity scores depending on the IPAT adjusted by group (support/no support) and the random intercept for practice and unit. These models were estimated both with and without the interaction between IPAT and group, i.e., enabling or disabling various slopes for the groups. The effect of support on the IPAT was assessed using the linear mixed effects model for the IPAT depending on group, time and the interaction between group and time adjusted by random intercept for cases, practice and unit with simple contrasts in the time domain. The interaction term in these models estimates the change of differences between groups from baseline. For all analyses the significant level was set to 0.05. Since the primary analysis considered only the effect of support on the IPAT, no adjustment for multiple testing was necessary.

Statistical analyses were conducted using SPSS 26.0 (IBM Corp., Armonk, NY) and R 4.0 [36], and MATLAB 2020b (MathWorks Inc., Natick, MA) was used for graphics.

## Results

### Sample, Implementation Process Assessment Tool (IPAT)-scores and fidelity scores

**Fig. 2:**
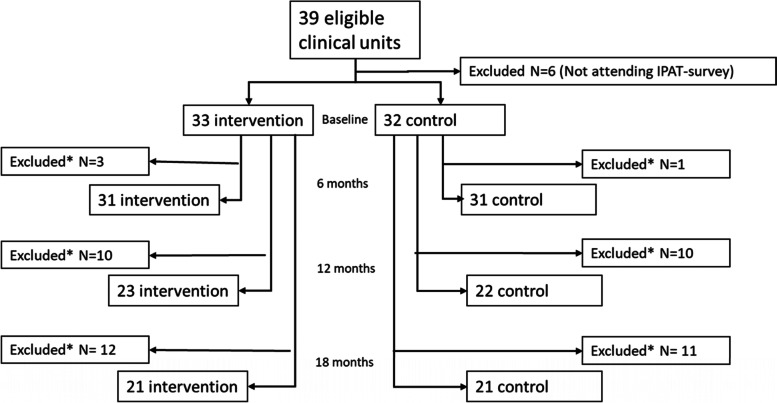
Flow diagram displaying the sample and exclusion during the 18-months period. *Clinical units were excluded for not meeting the inclusion criteria of a minimum of three responders on the IPAT questionnaire

Thirty-three of 39 clinical units were included. Following the inclusion criteria of three or more IPAT responders per unit, we excluded six units at baseline, eight at 6 months, sixteen at 12 months and eighteen at 18 months. Please see Fig. 2.

As shown in Table 1, the number of responses in the total sample decreased from 696 (response rate: 59%) at baseline to 326 (response rate: 30%) at eighteen months. After excluding responses missing data and those from units with less than three respondents, the valid sample consisted of 642 responses representing 33 units in the intervention arm and 32 in the control arm at baseline, decreasing to 298 responses representing 21 clinical units in both arms at eighteen months. The distribution of the IPAT respondents’ gender and profession reflects mental health care units in the present setting. In accordance with the inclusion criteria of “essential clinicians”, most respondents were trained clinical mental health care experts.

**Table 1:** Description of the responses and responders (each responded twice) on the IPAT scale at baseline, 6, 12 and 18 months

	Baseline	6 months*	12 months	18 months
Responses (response rate)	696 (59%)	464 (39%)	306 (27%)	326 (30%)
Excluded because of missing	45	16	0	0
Excluded because of less than three responses per unit	9	4	36	28
Included responses	**642**	**444**	**270**	**298**
Respondents per included clinic, mean (SD)	9.9 (4.0)	7.2 (4.6)	6.0 (2.1)	7.2 (3.2)
Gender				
Men	148 (23%)	89 (20%)	59 (22%)	66 (22%)
Women	494 (77%)	289 (65%)	211 (78%)	232 (78%)
Profession				
Medical doctor	51 (8%)	31 (7%)	22 (8%)	27 (9%)
Nurse	366 (57%)	222 (50%)	167 (62%)	194 (65%)
Psychologist	64 (10%)	31 (7%)	13 (5%)	18 (6%)
Social worker	45 (7%)	27 (6%)	19 (7%)	21 (7%)
Other	116 (18%)	67 (15%)	49 (18%)	39 (13%)
Specialist in mental health care				
Yes	404 (63%)	253 (57%)	194 (72%)	238 (80%)
No	238 (37%)	191 (43%)	76 (28%)	60 (20%)

*At 6 months 15% missing on description of the responders’ gender and profession. *SD* Standard Deviation

The mean IPAT scores per unit at baseline were higher for the intervention group (mean (CI) = 3.66 (3.45, 3.87)) than for the control group (mean (CI) = 3.31 (3.12, 3.49)). The fidelity scores were low for both the intervention arm (mean (CI) = 1.77 (1.49, 2.05)) and the control arm (mean (CI) = 1.82 (1.56, 2.07)). The IPAT and fidelity scores improved during the study period for both groups, yet a significantly larger improvement was seen where implementation support was provided than in the control arm (see Table 2).

**Table 2:** Mean fidelity scores and mean IPAT scores (mean (95% confidence interval)) per included unit for intervention (implementation support) and control arm at baseline, and at 6, 12 and 18 months

	Fidelity		Implementation Process Assessment Tool (IPAT)	
	*Supported*	*Control*	*Supported*	*Control*
Baseline (mean (CI)) (*N* = 33 for supported, *N* = 32 for control)	1.77 (1.49, 2.05)	1.82 (1.56, 2.07)	3.66 (3.45, 3.87)	3.31 (3.12, 3.49)
6 months (mean (CI)) (*N* = 31)	3.11 (2.74, 3.48)	1.86 (1.56, 2.16)	4.24 (3.98, 4.50)	3.72 (3.47, 3.97)
12 months (mean (CI)) (*N* = 23 for supported, *N* = 22 for control)	3.41 (2.90, 3.92)	2.02 (1.65, 2,39)	4.21 (3.88, 4.53)	3.54 (3.20, 3.88)
18 months (mean (CI)) (*N* = 21)	3.69 (3.15, 4.22)	2.38 (1.91, 2.86)	4.29 (3.96, 4.61)	3.44 (3.10, 3.77)

### Association between IPAT and fidelity

**Fig. 3:**
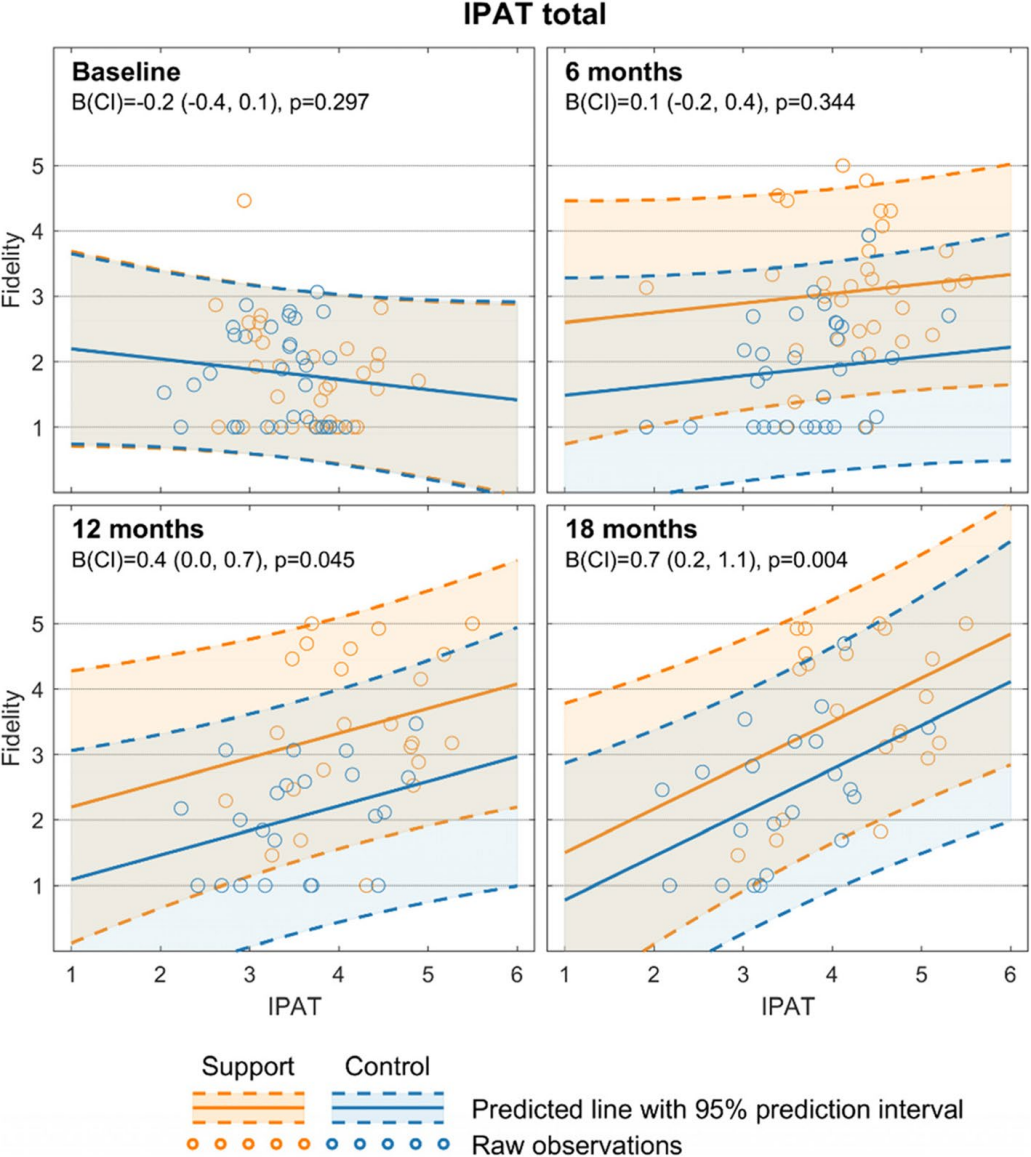
Correlations between the Implementation Process Assessment Tool score and fidelity score for the intervention and control groups every sixth month. Linear mixed model analysis model with time adjusted IPAT score as fixed effect and type of practice as random effect

As shown in Fig. 3, health care providers’ perceptions of the implementation effort (IPAT scores) and achieved implementation (fidelity score) were found to be significantly correlated at 12 and 18 months. The linear mixed effect model, adjusting for unit and practice, revealed a strong association at 18 months (B (CI) = .7 (0.2, 1.1) *p* = .004). At 12 months, the association was moderate (B (CI) = 0.4 (0.0, 0.7), *p* = .045). The analysis indicated no, or even negative, correlation at baseline; however, this was not significant.

### Implementation support’s impact on health professionals’ perceptions of the implementation effort

**Fig. 4:**
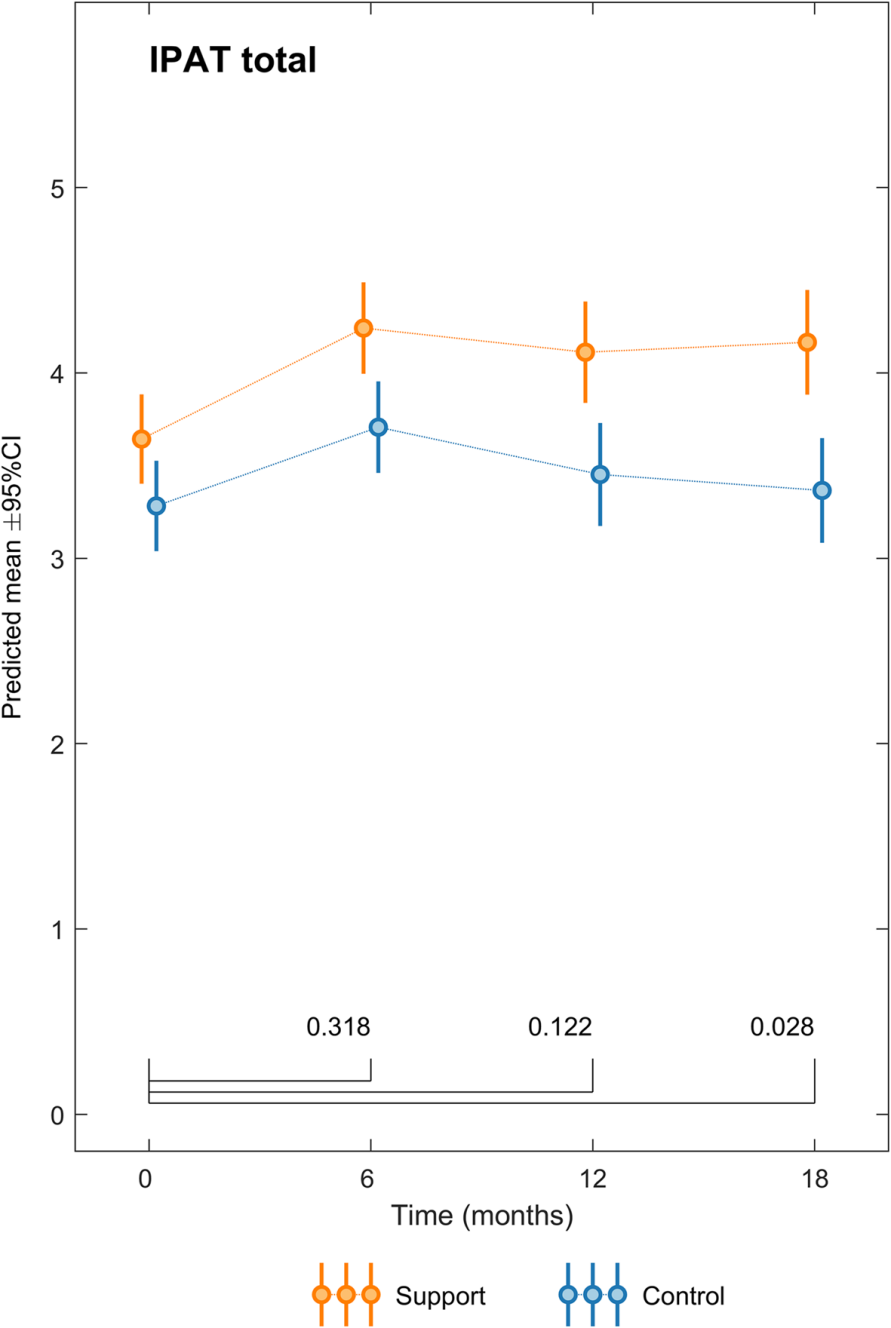
Comparison of IPAT scores between the intervention and control groups, adjusted for time and practice implemented, using a linear mixed model. Means and standard deviations (SD) are displayed in the figure (orange for intervention and blue for control group)

The health care providers reported higher scores on how they perceived the set of implementation factors when implementation support was provided than when it was not. Figure 4 displays the results of the linear mixed effect analysis, adjusting for time and type of practice implemented, confirming the positive association between implementation support (intervention) and the IPAT-score at 18 months (t(140) = 2.22, *p* = .028). The confidence intervals for the mean indicate significantly higher IPAT-scores in the intervention groups than in the control groups at all measurement times (see Table 2). Importantly, a difference in IPAT scores was present at baseline when the clinical units knew which practice they would receive support for.

## Discussion

In the present study, we found fidelity to be positively correlated with care providers’ perceptions of the implementation and that these perceptions can be affected by systematic implementation support. The correlations between fidelity and IPAT score were strongest late in the implementation process and were not present at baseline. The care providers perceived the implementation for the practice they received structured implementation support more positively than the control practice.

A relationship between how care providers perceive the implementation effort and implementation outcome, as we found, is in line with well-known theories and frameworks such as Readiness for change, Stages of change and CFIR [16, 19, 33, 37, 38]. However, we found the implementation factors and outcomes to be associated later in the implementation process and not at the onset. Our findings contrast with the literature emphasizing the investigation of facilitating factors only prior to an implementation effort [8, 19, 39]. They indicate that a more process-based approach to the readiness concept, as suggested by Stevens, among others [17], can be beneficial to understanding implementation. The changing IPAT scores during the 18-month period also suggest that health professionals assess the situation during all phases of the implementation to decide whether they will implement and sustain the new practice. This is in line with Nilsen, arguing that the progression of the implementation process and health professionals’ perceptions are mutually affected and that actions are mutually affected in a context [18, 40]. According to Stevens, the assumption that it is sufficient to establish readiness at baseline fails to understand the influence of context over time on individuals’ cognitive and affective evaluations and responses [17]. Furthermore, our results are in line with complex intervention theory highlighting mutual interactions between intervention and context factors [10] and with evidence for repeated feedback’s impact on maintaining engagement during the implementation process [41]. Our results suggest that care providers’ perceptions and implementation outcomes are mutually affected, creating a positive or negative spiral of improvement success or failure, as described by Øvretveit [42].

The implementation literature states that systematically developed intervention bundles can affect health professionals’ engagement and implementation outcomes [33, 43]. The intervention in the present study was designed to affect the same construct of care providers’ individual and collective readiness and engagement as measured by the IPAT questionnaire. It combines several recommended interventions, such as continuous measurement and provision of feedback, clinical training, implementation training and team support [1, 43–45]. Our finding of a significantly higher score on the IPAT scale when implementation support was provided than when it was not was therefore expected. What we believe to be innovative is the measurement of and feedback on how the implementers experience the implementation effort. Responding to the questionnaire may introduce reflections on effective implementation among the responders, and the manager can gain insight into the facilitation needs. We combined this with guidance from implementation facilitators trained to support team activities to meet the needs revealed by the IPAT scores. Tailoring implementation support to the present needs of the implementers is emphasized in the literature [1]; however, we cannot state which part of the intervention was the most effective. Interestingly, we also found the IPAT score to be higher for the practices the clinics knew they would receive support for before the onset of systematic implementation support. We believe this is best understood by readiness theories recognizing the importance of self-induced initial preparation and collective tuning in on the forthcoming implementation effort [46].

Few implementation factors are tested for their associations with implementation outcomes. The review by Chaudoir et al. found only one study that investigate explanations for variation in fidelity were investigated [7]. In the present study, we employed a questionnaire based on a combination of theories [32]. Given the complexity of implementation, we believe that the extraction of prominent factors from a set of theories is more likely to explain implementation than scales based on single theories. The significant correlation we found between the IPAT score and fidelity indicates that the questionnaire is well suited for investigating the “why” in implementation.

The present study used a cluster-randomized longitudinal design including a large sample of clinical units followed for 18 months. We gathered data four times on implementation factors and -outcomes for both the intervention and the control arms. Most investigations of the implementation process have used a retrospective design and/or qualitative methods, implying a risk of bias related to the informants’ and researchers’ recollection and insight into the implementation process [2, 47]. The repeated measurement we conducted every sixth month enabled an investigation of associations between perceptions and actual change to practice in various phases of the process that qualitative or pre-post design cannot. The design, where each clinical unit was assigned to both intervention and control arms for two practices they had chosen to implement, reduced the risk of bias. However, there is a risk of spillover effects from the intervention to the control arm. The intervention included quality improvement training and methods expected to be useful in most implementation efforts. The improved fidelity scores in the control arm may indicate that the clinics were to some degree able to adapt these methods for the control practice. If so, it should be seen as a strength of the tested intervention that it can provide clinics with implementation competence that is transferable to other implementation efforts. However, for research purposes, it implies that we may underestimate the effect of the intervention.

## Limitations

Concerns regarding response rates, statistical power and potential confounding factors should be considered when interpreting the results of the present study. The response rates decreased during the 18-month period. We do not know why the response rates decreased or to what degree the mean score per clinic represents the invited sample, but the distribution of represented professions lends support for representability. Implementing four practices measured by four unique fidelity scales reduces the statistical power compared to investigating only one or two practices, implying a risk of type II error (not detecting an existing association). The IPAT scores were higher in the intervention group than in the control group at baseline. This may indicate that the clinics gave priority to the practice they knew they would receive support for and were able to self-induce steps to improve care providers’ readiness before onset of the defined intervention. The impact of preparatory steps is of great interest for understanding implementation, but was not defined in the study as a part of the intervention and therefore adjusted for in the analysis. Retrospectively, we can see that we underestimated the impact of the minor initial preparatory steps we performed. No measures were taken to limit engagement in the control practice, which may have reduced the differences between the intervention and control arms. Further, we did not systematically investigate how each clinic employed the various elements of the implementation support. Given the strong evidence for the clinical practices that were implemented, we believe implementation success (programme fidelity) can constitute a valid intermediate outcome for patient outcomes; however, this was not investigated in the present study.

Each clinic chose two of four practices. Logically, we expect the clinics to choose practices they need to implement. The generally low score initially indicates that this was true. Our results and conclusions should be interpreted as true when implementing a practice that the clinical unit believes to be beneficial for them. We do not know the degree to which our results are valid for situations in which clinical units are obliged to implement a practice they have not chosen themselves. We have also not investigated the degree to which our results are true for each of the four subgroups of practices. Potential differences between the practices, such as different levels of clinical supervision, was not investigated.

The study was conducted in Norwegian specialist mental health care and regards the implementation of evidence-based practices for patients suffering from psychosis. The sample represents typical multiprofessional clinical units from urban and rural areas of Norway. The IPAT is based on international acknowledged literature and is expected to be valid for similar Western health services. It was developed for health care services in general but has been tested only within mental health care. The generalizability of the present study’s results and conclusions to health services for other patient groups or to other countries is unknown.

## Conclusion

The present study indicates that implementation can be facilitated by improving how care providers perceive the situation of implementation and that these perceptions can be improved by systematic implementation support. The results suggest less emphasis on preparedness prior to implementation and more emphasis on care providers’ continuous assessment of pros and cons for implementation and how we tailor the support to meet these changing needs. The stronger association of implementation factors and implementation outcomes with larger differences between the intervention and control groups at 18 months after the onset of implementation support highlight the importance of long-term planning and facilitation to stabilize the new practice. However, further research is needed to understand how we can provide efficient implementation support. New studies should investigate whether some implementation factors are more important than others at various phases of the implementation process and whether our results can be generalized to the implementation of other practices and in other health services.

## Supplementary Information

**Additional file 1. Supplementary table**. Number of responses per clinic at baseline, 6, 12 and 18 months, displayed by practices and intervention or control arms.

## Data Availability

The datasets generated and analysed during the current study are not publicly available due to ongoing investigations using the same dataset but are available from the corresponding author on reasonable request.

## Abbreviations

IPAT: Implementation process assessment tool (questionnaire)
IMR: Illness management and recovery (practice)
FAM: Family psychoeducation (practice)
MED: Anti-psychotic medication (practice)
PHYS: Physical health care (practice)

## References

[1] Albers B, Shlonsky A, Mildon R. Implementation Science 3.0: Springer International Publishing; 2020. 10.1007/978-3-030-03874-8.

[2] Nilsen P, Birken SA. Handbook on Implementation Science: Edward Elgar publishing; 2020. 10.4337/9781788975995.

[3] Carroll C, Patterson M, Wood S, Booth A, Rick J, Balain S. A conceptual framework for implementation fidelity. Implement Sci. 2007;2:40. 10.1186/1748-5908-2-40. 10.1186/1748-5908-2-40 PMC2213686 18053122

[4] Proctor E, Silmere H, Raghavan R, Hovmand P, Aarons G, Bunger A, Griffey R, Hensley M. Outcomes for implementation research: conceptual distinctions, measurement challenges, and research agenda. Adm Policy Ment Health. 2011;38(2):65–76. 10.1007/s10488-010-0319-7. 10.1007/s10488-010-0319-7 PMC3068522 20957426

[5] Kelly P, Hegarty J, Barry J, Dyer KR, Horgan A. A systematic review of the relationship between staff perceptions of organizational readiness to change and the process of innovation adoption in substance misuse treatment programs. J Subst Abuse Treat. 2017;80:6–25. 10.1016/j.jsat.2017.06.001. Epub 2017 Jun 1. 10.1016/j.jsat.2017.06.001 28755775

[6] Torrey WC, Bond GR, McHugo GJ, et al. Evidence-Based Practice Implementation in Community Mental Health Settings: The Relative Importance of Key Domains of Implementation Activity. Admin Pol Ment Health. 2012;39:353–64. 10.1007/s10488-011-0357-9. 10.1007/s10488-011-0357-9 21574016

[7] Chaudoir SR, Dugan AG, Barr CH. Measuring factors affecting implementation of health innovations: a systematic review of structural, organizational, provider, patient, and innovation level measures. Implement Sci. 2013;8:22. Published 2013 Feb 17. 10.1186/1748-5908-8-22. 10.1186/1748-5908-8-22 PMC3598720 23414420

[8] Helfrich CD, Li YF, Sharp ND, Sales AE. Organizational readiness to change assessment (ORCA): development of an instrument based on the Promoting Action on Research in Health Services (PARIHS) framework. Implement Sci. 2009;4:38. Published 2009 Jul 14. 10.1186/1748-5908-4-38. 10.1186/1748-5908-4-38 PMC2716295 19594942

[9] Weiner BJ, Lewis CC, Stanick C, Powell BJ, Dorsey CN, Clary AS, Boynton MH, Halko H. Psychometric assessment of three newly developed implementation outcome measures. Implement Sci. 2017;12(1):108. 10.1186/s13012-017-0635-3. 10.1186/s13012-017-0635-3 PMC5576104 28851459

[10] Richards DA, Rahm Hallberg I (2015). Complex Interventions in Health: An overview of research methods.

[11] Craig P, Dieppe P, Macintyre S, et al. Developing and evaluating complex interventions: the new Medical Research Council guidance. BMJ. 2008;337:a1655. Published 2008 Sep 29. 10.1136/bmj.a1655. 10.1136/bmj.a1655 PMC2769032 18824488

[12] Shea CM, Jacobs SR, Esserman DA, Bruce K, Weiner BJ. Organizational readiness for implementing change: a psychometric assessment of a new measure. Implement Sci. 2014;9:7. Published 2014 Jan 10. 10.1186/1748-5908-9-7. 10.1186/1748-5908-9-7 PMC3904699 24410955

[13] Aarons GA. Mental health provider attitudes toward adoption of evidence-based practice: the Evidence-Based Practice Attitude Scale (EBPAS). Ment Health Serv Res. 2004;6(2):61–74. 10.1023/b:mhsr.0000024351.12294.65. 10.1023/b:mhsr.0000024351.12294.65 PMC1564126 15224451

[14] Rogers EM (1995). Diffusion of innovations (4ed).

[15] Grol R, Wensing M. What drives change? Barriers to and incentives for achieving evidence-based practice. Med J Aust. 2004;180(S6):S57–60. 10.5694/j.1326-5377.2004.tb05948.x. 10.5694/j.1326-5377.2004.tb05948.x 15012583

[16] Prochaska JO, DiClemente CC. Transtheoretical therapy: Toward a more integrative model of change. Psychotherapy: Theory, Research & Practice. 1982;19(3):276–88. 10.1037/h0088437.

[17] Stevens GW. Toward a process-based approach of conceptualizing change readiness. J Appl Behav Sci. 2013;49(3):333–60. 10.1177/0021886313475479.

[18] Nilsen P, Bernhardsson S. Context matters in implementation science: a scoping review of determinant frameworks that describe contextual determinants for implementation outcomes. BMC Health Serv Res. 2019;19(1):189. Published 2019 Mar 25. 10.1186/s12913-019-4015-3. 10.1186/s12913-019-4015-3 PMC6432749 30909897

[19] Kaplan HC, Provost LP, Froehle CM, Margolis PA. The Model for Understanding Success in Quality (MUSIQ): building a theory of context in healthcare quality improvement. BMJ Qual Saf. 2012;21(1):13–20. 10.1136/bmjqs-2011-000010. Epub 2011 Aug 10. 10.1136/bmjqs-2011-000010 21835762

[20] Chamberlain P, Brown CH, Saldana L. Observational measure of implementation progress in community based settings: the Stages of Implementation Completion (SIC). Implement Sci. 2011;6:116. 10.1186/1748-5908-6-116. 10.1186/1748-5908-6-116 PMC3197550 21974914

[21] Argyris C (1977). Double loop learning in organizations. Harv Bus Rev.

[22] Pereira VC, Silva SN, Carvalho VKS, Zanghelini F, Barreto JOM. Strategies for the implementation of clinical practice guidelines in public health: an overview of systematic reviews. Health Res Policy Syst. 2022;20(1):13. 10.1186/s12961-022-00815-4. 10.1186/s12961-022-00815-4 PMC8785489 35073897

[23] Lloyd-Evans B, Osborn D, Marston L (2020). The CORE service improvement programme for mental health crisis resolution teams: Results from a cluster-randomised trial. Br J Psychiatry.

[24] Williams NJ, Beidas RS. Annual Research Review: The state of implementation science in child psychology and psychiatry: a review and suggestions to advance the field. J Child Psychol Psychiatr. 60:430–50. 10.1111/jcpp.12960. 10.1111/jcpp.12960 PMC6389440 30144077

[25] McHugo GJ, Drake RE, Whitley R, Bond GR, Campbell K, Rapp CA, Goldman HH, Lutz WJ, Finnerty MT. Fidelity outcomes in the National Implementing Evidence-Based Practices Project. Psychiatr Serv. 2007;58(10):1279–84. 10.1176/ps.2007.58.10.1279. 10.1176/ps.2007.58.10.1279 17914003

[26] Ruud T, Drake RE, Šaltytė Benth J, et al. The Effect of Intensive Implementation Support on Fidelity for Four Evidence-Based Psychosis Treatments: A Cluster Randomized Trial. Adm Policy Ment Health. 2021;48(5):909–20. 10.1007/s10488-021-01136-4. 10.1007/s10488-021-01136-4 PMC8363529 33871742

[27] Hempel S, O'Hanlon C, Lim YW, Danz M, Larkin J, Rubenstein L. Spread tools: a systematic review of components, uptake, and effectiveness of quality improvement toolkits. Implement Sci. 2019;14(1):83. 10.1186/s13012-019-0929-8. 10.1186/s13012-019-0929-8 PMC6701087 31426825

[28] Ruud T, Høifødt TS, Hendrick DC, Drake RE, Høye A, Landers M, Heiervang KS, Bond GR. The Physical Health Care Fidelity Scale: Psychometric Properties. Adm Policy Ment Health. 2020;47(6):901–10. 10.1007/s10488-020-01019-0. 10.1007/s10488-020-01019-0 PMC7547955 32036479

[29] Ruud T, Drivenes K, Drake RE, Haaland VØ, Landers M, Stensrud B, Heiervang KS, Tanum L, Bond GR. The Antipsychotic Medication Management Fidelity Scale: Psychometric properties. Adm Policy Ment Health. 2020;47(6):911–9. 10.1007/s10488-020-01018-1. 10.1007/s10488-020-01018-1 PMC7547997 32030595

[30] Joa I, Johannessen JO, Heiervang KS, Sviland AA, Nordin HA, Landers M, Ruud T, Drake RE, Bond GR. The Family Psychoeducation Fidelity Scale: Psychometric Properties. Adm Policy Ment Health. 2020;47(6):894–900. 10.1007/s10488-020-01040-3. 10.1007/s10488-020-01040-3 PMC7547979 32323217

[31] Egeland KM, Heiervang KS, Landers M, Ruud T, Drake RE, Bond GR. Psychometric Properties of a Fidelity Scale for Illness Management and Recovery. Adm Policy Ment Health. 2020;47(6):885–93. 10.1007/s10488-019-00992-5. 10.1007/s10488-019-00992-5 PMC7547988 31701294

[32] Hartveit M, Hovlid E, Nordin MHA, et al. Measuring implementation: development of the implementation process assessment tool (IPAT). BMC Health Serv Res. 2019;19(1):721. Published 2019 Oct 21. 10.1186/s12913-019-4496-0. 10.1186/s12913-019-4496-0 PMC6805659 31638967

[33] Damschroder LJ, Aron DC, Keith RE, Kirsh SR, Alexander JA, Lowery JC. Fostering implementation of health services research findings into practice: a consolidated framework for advancing implementation science. Implement Sci. 2009;4:50. 10.1186/1748-5908-4-50. 10.1186/1748-5908-4-50 PMC2736161 19664226

[34] Weiner BJ, Amick H, Lee SY. Conceptualization and measurement of organizational readiness for change: a review of the literature in health services research and other fields. Med Care Res Rev. 2008;65(4):379–436. 10.1177/1077558708317802. Epub 2008 May 29. 10.1177/1077558708317802 18511812

[35] Grol R, Wensing M, Eccles M, Davis D. Improving patient care: the implementation of change in health care (2ed). Oxford: John Wiley & Sons; 2013.

[36] Team RC (2013). R: A language and environment for statistical computing.

[37] Fixsen DL, Naoom SF, Blase K, Fiedman RM, Wallace F (2005). Implementation research: a synthesis of the literature.

[38] Weiner BJ. A theory of organizational readiness for change. Implement Sci. 2009;4:67. 10.1186/1748-5908-4-67. 10.1186/1748-5908-4-67 PMC2770024 19840381

[39] Egeland KM, Ruud T, Ogden T, Lindstrøm JC, Heiervang KS. Psychometric properties of the Norwegian version of the Evidence-Based Practice Attitude Scale (EBPAS): to measure implementation readiness. Health Res Policy Syst. 2016;14(1):47. 10.1186/s12961-016-0114-3. 10.1186/s12961-016-0114-3 PMC4912744 27316675

[40] Nilsen P. Making sense of implementation theories, models and frameworks. Implement Sci. 2015;10:53. Published 2015 Apr 21. 10.1186/s13012-015-0242-0. 10.1186/s13012-015-0242-0 PMC4406164 25895742

[41] Ovretveit J, Mittman B, Rubenstein L, Ganz DA. Using implementation tools to design and conduct quality improvement projects for faster and more effective improvement. Int J Health Care Qual Assur. 2017;30(8):755–68. 10.1108/IJHCQA-01-2017-0019. 10.1108/IJHCQA-01-2017-0019 28958203

[42] Øvretveit J (1990). Quality health services.

[43] Greenhalgh T. How to implement evidence-based healthcare: John Wiley & Sons; 2017.

[44] Borgert MJ, Goossens A, Dongelmans DA. What are effective strategies for the implementation of care bundles on ICUs: a systematic review. Implement Sci. 2015;10:119. Published 2015 Aug 15. 10.1186/s13012-015-0306-1. 10.1186/s13012-015-0306-1 PMC4536788 26276569

[45] Ritchie MJ, Kirchner JE, Parker LE, et al. Evaluation of an implementation facilitation strategy for settings that experience significant implementation barriers. Implement Sci. 2015;10(Suppl 1):A46. Published 2015 Aug 20. 10.1186/1748-5908-10-S1-A46.

[46] Holt DT, Vardaman JM. Toward a comprehensive understanding of readiness for change: The case for an expanded conceptualization. J ChangManag. 2013;13(1):9–18. 10.1080/14697017.2013.768426.

[47] Brownson RC, Colditz GA, Proctor EK. Dissemination and implementation research in health: translating science to practice (2ed). New York: Oxford University Press; 2018.

