# Supplementary material for: Can systematic implementation support improve programme fidelity by improving care providers’ perceptions of implementation factors? A cluster randomized trial
Source: BMC Health Serv Res. 2022 Jun 22;22:808. doi: 10.1186/s12913-022-08168-y (PMC9215018; doi:10.1186/s12913-022-08168-y)
Supplement: Supplementary file 1 — Additional file 1. Supplementary table. Number of responses per clinic at baseline, 6, 12 and 18 months, displayed by practices and intervention or control arms. [file 12913_2022_8168_MOESM1_ESM.docx]

Supplementary table: Number of responses per clinic at baseline, 6, 12 and 18 months, displayed by practices and intervention or control arms.

|  |  | Unit | Baseline | 6 months | 12 months | 18 months |
| --- | --- | --- | --- | --- | --- | --- |
| Physical health care practice  (PHYS) | Intervention | 1a | 14 | 18 | - | - |
|  |  | 1b | 5 | - | - | - |
|  |  | 1c | 13 | 10 | 4 | - |
|  |  | 3a | 10 | 5 | 4 | 12 |
|  |  | 3b | 9 | 9 | 9 | 9 |
|  |  | 3c | 9 | 4 | 3 | - |
|  |  | 4a | 6 | 4 | 4 | 4 |
|  |  | 4b | 3 | - | - | 3 |
|  |  | 5a | 24 | 22 | 6 | 17 |
|  |  | 6a | 13 | 12 | 11 | 11 |
|  |  | 6b | 9 | 6 | 6 | 6 |
|  |  | 6c | 8 | 4 | 6 | 8 |
|  | Control | 1d | 8 | 3 | - | - |
|  |  | 1e | 3 | 3 | - | - |
|  |  | 1f | 8 | - | - | - |
|  |  | 1g | 8 | 5 | - | - |
|  |  | 2a | 6 | 5 | 7 | 8 |
|  |  | 3d | 6 | 3 | 3 | - |
|  |  | 3e | 8 | 6 | 5 | 6 |
|  |  | 3f | 20 | 14 | 10 | - |
|  |  | 4c | 10 | 8 | 6 | 4 |
|  |  | 4d | 12 | 4 | 4 | 6 |
|  |  | 4e | - | 3 | 7 | 7 |
|  |  | 6d | 9 | 8 | 5 | 5 |
|  |  | 6e | 11 | 6 | 6 | 8 |
| Illness management and  recovery program (IMR) | Intervention | 1h | 8 | 5 | - | - |
|  |  | 2a | 8 | 5 | 7 | 8 |
|  |  | 2b | 11 | 9 | 8 | 7 |
|  |  | 2c | 9 | 7 | 8 | 5 |
|  |  | 3e | 8 | 6 | 5 | 6 |
|  |  | 4d | 12 | 6 | 4 | 6 |
|  |  | 4e | - | 3 | 7 | 7 |
|  |  | 4h | 5 | 3 | - | 4 |
|  |  | 5b | 11 | 3 | 4 | 4 |
|  |  | 6d | 11 | 9 | 5 | 5 |
|  | Control | 3a | 11 | 5 | 4 | 12 |
|  |  | 3b | 11 | 10 | 9 | 9 |
|  |  | 3c | 12 | 4 | 3 | - |
|  |  | 4f | 12 | 9 | 6 | 5 |
|  |  | 6a | 13 | 13 | 11 | 11 |
|  |  | 6b | 9 | 6 | 6 | 6 |
|  |  | 6c | 9 | 5 | 6 | 8 |
|  |  | 6f | 9 | 3 | - | - |
|  |  | 6g | 17 | 16 | 6 | 8 |
| Antipsychotic medication  Management (MED) | Intervention | 1d | 8 | 3 | - | - |
|  |  | 1e | 3 | 3 | - | - |
|  |  | 1i | 10 | 5 | - | - |
|  |  | 3d | 18 | 9 | 10 | - |
|  |  | 6e | 10 | 6 | 6 | 8 |
|  |  | 6f | 6 | 3 | - | - |
|  | Control | 1a | 12 | 16 | - | - |
|  |  | 1c | 13 | 10 | 4 | - |
|  |  | 2c | 8 | 12 | 8 | 5 |
|  |  | 4g | - | - | - | 3 |
|  |  | 5a | 21 | 21 | 6 | 17 |
|  |  | 5b | 11 | 3 | - | 4 |
| Family psychoeducation program  (FAM) | Intervention | 1f | 8 | - | - | - |
|  |  | 1g | 8 | 5 | - | - |
|  |  | 3d | 6 | 3 | 3 | - |
|  |  | 4c | 10 | 9 | 6 | 8 |
|  |  | 4f | 11 | 9 | 4 | 5 |
|  |  | 6g | 16 | 14 | 6 | 8 |
|  | Control | 1b | 6 | - | - | - |
|  |  | 1h | 8 | 5 | - | - |
|  |  | 1i | 10 | 5 | - | - |
|  |  | 2b | 10 | 7 | 8 | 7 |
|  |  | 4a | 6 | 4 | 4 | 4 |
|  |  | 4h | 5 | 3 | - | 4 |

The clinics are coded by a number representing the local health trust in which they are organised, and a letter representing their unique clinic.

“-“ = less than three responses at the time of measuring are excluded from the study.
